# Safety and Efficacy of PD-1/PD-L1 Inhibitors in Cancer Patients With Preexisting Autoantibodies

**DOI:** 10.3389/fimmu.2022.893179

**Published:** 2022-05-16

**Authors:** Hui Tang, Ruixuan Geng, Xiuxiu Xu, Yingyi Wang, Jiaxin Zhou, Shulan Zhang, Lin Zhao, Mei Guan, Chunmei Bai

**Affiliations:** ^1^ Department of Medical Oncology, Peking Union Medical College Hospital, Chinese Academy of Medical Sciences and Peking Union Medical College, Beijing, China; ^2^ Department of International Medical Services, Peking Union Medical College Hospital, Chinese Academy of Medical Sciences and Peking Union Medical College, Beijing, China; ^3^ Department of Rheumatology and Clinical Immunology, Peking Union Medical College Hospital, Chinese Academy of Medical Sciences and Peking Union Medical College, National Clinical Research Center for Dermatologic and Immunologic Diseases (NCRC-DID), Ministry of Science and Technology; Key Laboratory of Rheumatology and Clinical Immunology, Ministry of Education, Beijing, China

**Keywords:** programmed cell death-1, antinuclear antibody, anti-Ro52 antibody, antithyroid antibody, immune-related adverse events

## Abstract

**Background:**

Programmed cell death protein-1/programmed cell death ligand-1 (PD-1/PD-L1) inhibitors therapy is now a routine scheme in cancers. However, the effect of preexisting autoantibodies on the safety and efficacy of PD-1/PD-L1 inhibitors in cancer patients is not well understood.

**Methods:**

The present retrospective cohort study evaluated the safety and efficacy of PD-1/PD-L1 inhibitors in patients with preexisting autoantibodies. Patients who received PD-1/PD-L1 inhibitors in the Department of Medical Oncology, Peking Union Medical College Hospital between November 2017 and August 2021 were reviewed.

**Results:**

67 (37.9%) of the 177 patients, 27 (20.3%) of the 133 patients, and 16 (11.0%) of 146 patients who received PD-1/PD-L1 inhibitors were positive for ANA, anti-Ro52, and antithyroid antibodies, respectively. Preexisting ANA and anti-Ro52 antibody were not associated with the increased risk of immune-related adverse events (irAEs), while thyroid dysfunction was more frequent in patients with positive antithyroid antibody (75.0% versus 13.8%, p < 0.001). The median progression-free survival (PFS, 13.1 versus 7.0 months, p = 0.015) was significantly longer in the ANA-positive patients, while the median overall survival (OS, 14.5 versus 21.8 months, p = 0.67) did not differ significantly between the ANA-positive and ANA-negative groups. Moreover, the preexisting anti-Ro52 and antithyroid antibodies were not significantly associated with PFS and OS.

**Conclusions:**

The presence of ANA and anti-Ro52 antibody were not associated with a higher risk of irAEs, whereas patients positive for antithyroid antibody should monitor closely immune-related thyroid dysfunction. Preexisting ANA might be a predictor of longer PFS, while anti-Ro52 and antithyroid antibodies had no significant effect on survival outcomes in patients receiving PD-1/PD-L1 inhibitors therapy.

## Introduction

Immune checkpoint inhibitors (ICIs), especially monoclonal antibodies targeting the PD-1 (programmed cell death-1)–PD-L1 (programmed cell death-ligand 1) axis, have improved outcomes for a variety of malignancies (1). ICIs work *via* breaking the state of immune tolerance in the tumor microenvironment, resulting in robust activation of the immune system and subsequent antitumor immune response (1). However, enhanced T cell activation may cause immune-related adverse events (irAEs), which occur approximately in 40%–50% of patients treated by ICIs (2). Therefore, it is important to identify patients who are more likely to develop irAEs or respond to ICIs.

Antinuclear antibody (ANA) profile is a spectrum of heterogeneous autoantibodies against various nuclear and cytoplasmic components (3). Given that ANA positivity may indicate a predisposition to immune activation, it is understandable that some clinicians are concerned that patients positive for ANA may be at a higher risk of irAEs (4). However, the effect of ANA on the safety and efficacy of ICIs in cancer patients is still controversial (5–9). Moreover, antithyroid antibody was suggested to be associated with thyroid dysfunction after ICIs treatment (6). Anti-Ro52 (TRIM21) antibody, one member of the ANA profile, is regarded to be associated with many autoimmune diseases, especially Sjogren’s syndrome, systemic lupus erythematosus, and systemic sclerosis (10). The prevalence of anti-Ro52 antibody varies in malignant diseases (11, 12). Previous studies suggested that anti-Ro52 positivity was correlated with better overall survival in patients with ovarian cancer (11). Whether the presence of anti-Ro52 antibody might affect the safety or efficacy of ICIs has remained unknown. Thus, the present retrospective cohort study aimed to evaluate the safety and efficacy of PD-1/PD-L1 inhibitors in cancer patients with preexisting autoantibodies.

## Materials and Methods

### Patients

Between November 2017 and August 2021, the data of patients with cancer who received PD-1/PD-L1 inhibitors in the Department of Medical Oncology, Peking Union Medical College Hospital (PUMCH) was obtained from the hospital’s medical records. Inclusion criteria were as follows: 1) patients with histopathologically confirmed cancers; 2) received at least 1 cycle of PD-1/PD-L1 inhibitor therapy; 3) ANA test completed within one month before immunotherapy initiation was available. The exclusion criteria were as follows: 1) loss of follow-up within one month after the initiation of immunotherapy; 2) survival outcomes or irAEs could not be assessed; 3) Combined with secondary primary tumors that may affect patients’ survival outcomes, and confound the efficacy or irAEs evaluation. This study was approved by the Medical Ethics Committee of PUMCH (S-K1949). Patients’ consents for participation and publication were waived by the Medical Ethics Committee due to the retrospective design and the deidentified data of this study.

### Assessments

Testing results of ANA, ANA profile, antithyroglobulin, and antithyroid peroxidase within one month before immunotherapy initiation were screened. ANA profile was determined by line immunoassay (Euroimmun, Lubeck, Germany), which consists of autoantibodies against antigens including Ro52, SSA, SSB, dsDNA, Sm, rRNP, U1RNP, Scl-70, PM-Scl, Jo-1, CENP-B, PCNA, nucleosomes, mitochondrial M2, and Histones. ANA titer was measured by indirect immunofluorescence assay using Hep-2 cells (Euroimmun, Lubeck, Germany), while antithyroglobulin and antithyroid peroxidase were determined by the Siemens Centaur XP Chemiluminescent Immunoassay platform (Siemens, Ireland) with the antithyroglobulin antibody IgG (Siemens, Cat. No. 10492399, USA) and the thyroid peroxidase antibody IgG (Siemens, Cat. No. 10630887, USA) (13). Patients with ANA titers ≥ 1:80 were considered ANA-positive (14). Moreover, those were considered positive for antithyroid antibody if either antithyroglobulin or antithyroid peroxidase was positive. Those were considered positive for any preexisting antibody if all autoantibodies mentioned above were examined and any autoantibody mentioned above was positive. The level of LDH, IgG, IgA, IgM, hsCRP were determined by a commercial nephelometry assay using AU series clinical chemistry analyzer (Beckman Coulter, Brea, USA). ESR were measured by VACUETTE^®^ Automated ESR Systems (Greiner Bio-One, Kremsmünster, Austria). The level of IL-6, IL-8, IL-10, TNF-α were determined by Chemiluminescent Immunoassay using the IMMULITE^®^ 1000 system (Siemens, Erlangen, Germany). Serum free triiodothyronine (FT3), free thyroxine (FT4), thyroglobulin (Tg), and thyroid-stimulating hormone (TSH) levels were determined by chemiluminescence immunoassay (Roche Diagnostics, Germany).

PD-1/PD-L1 inhibitor therapy was provided until tumor progression or unacceptable toxicity was noted. All patients were followed up until death or loss of contact, with a follow-up deadline of January 2022. The irAEs severity was graded according to the Common Terminology Criteria for Adverse Events version 5.0. To examine thyroid dysfunction, the serum levels of FT3, FT4, Tg, and TSH were assessed at baseline and every 6 weeks during immunotherapy administration. Thyroid dysfunction or irAE was defined as described in the previously published study (15). Briefly, newly developed abnormal FT3, FT4, Tg, and TSH levels, with or without new or significant exacerbation of symptoms of hyperthyroidism/hypothyroidism, were regarded as thyroid dysfunction. All patients were evaluated by computed tomography (CT) scans or magnetic resonance imaging (MRI) every 6 to 12 weeks. Progression-free survival (PFS) and overall survival (OS) were measured as the time from immunotherapy onset to tumor progression or death due to any cause (PFS) or to the latter (OS). Tumor response was evaluated based on the Response Evaluation Criteria in Solid Tumors version 1.1 (16). The objective response rate (ORR) was defined as the proportion of patients who had a complete or partial response to therapy, whereas the disease control rate (DCR) was defined as the proportion of patients who had a complete or partial response to therapy or stable disease.

### Statistical Analysis

The Mann-Whitney U tests were used to compare continuous variables between two groups. Chi-squared and Fisher’s exact tests were used to examine the correlation between two categorical variables. Survival outcome was evaluated by the Kaplan-Meier method and was compared between groups using the log-rank test. Additionally, propensity-score matching (PSM) was utilized to minimize the impact of confounding factors. The propensity scores were calculated based on age, TNM stage, cancer type, and Eastern Cooperative Oncology Group (ECOG) performance status (PS) score. Furthermore, least absolute shrinkage and selection operator (LASSO) regression analysis was conducted to prevent collinearity among the candidate indicators of survival outcomes. Furthermore, univariate and multivariate Cox proportional hazard models were performed to calculate the hazard ratios (HR) with 95% confidence intervals (CI) of variables associated with survival outcomes in patients. Only variables, which significantly associated with survival outcome in univariate analysis, will be included in multivariate analysis. All statistical analyses and visualization were performed using R software (version 3.6.1, https://www.r-project.org/). A two-tailed value of P < 0.05 was considered to statistically significant.

## Result

### Patient Characteristics

Of the 177 enrolled patients with available ANA testing result, the main tumor types were digestive tract cancers and non-small cell lung cancer (NSCLC). The median age was 61 (range, 22-85) years, 172 patients (97.2%) had an ECOG PS score of 0 or 1, 149 (84.2%) had stage IV disease, and 98 (55.4%) experienced no prior systemic anti-cancer therapy (**Table 1**). Of all enrolled patients, 91 (51.4%) had at least one positive autoantibody, 67 (37.9%) were positive for ANA. Among 111 patients who completed ANA, ANA profile, and antithyroid antibody tests before immunotherapy initiation, 66 (59.5%) had at least one positive autoantibody (we defined this as positive for any preexisting antibody). Moreover, among 146 patients who completed antithyroid antibody tests before immunotherapy initiation, 16 (11.0%) patients were positive for antithyroid antibody (either antithyroglobulin or antithyroid peroxidase was positive). Among the members of ANA profile, the most common autoantibody was anti-Ro52 antibody (27/133, 20.3%), and the positive rate of any other autoantibody was less than 6%. In particular, 3 patients were previously diagnosed with autoimmune diseases before immunotherapy, including 1 each with immune thrombocytopenia, Hashimoto’s thyroiditis, and vitiligo. At the time of immunotherapy initiation, no patient had active autoimmune diseases. Moreover, no patient had newly developed autoimmune diseases during immunotherapy. At the time of analysis, the median follow-up duration was 8.2 (range, 0.4-36) months.

**Table 1:** Baseline characteristics of patients with or without preexisting antibodies.

Variables	Positive ANA	Negative ANA	P value	Positive for any preexisting antibody	Negative for all preexisting antibodies	P value
	(n=67)	(n=110)		(n=66)	(n=45)	
Age, median (range), years	62 (32-81)	61 (22-85)	0.478	59 (32, 83)	58 (32, 85)	0.694
Sex, male	44 (65.7%)	80 (72.7%)	0.409	46 (69.7%)	35 (77.8%)	0.469
Tumor type						
NSCLC	16 (23.9%)	30 (27.3%)	**0.020**	18 (27.3%)	9 (20.0%)	**0.026**
GC	13 (19.4%)	19 (17.3%)		8 (12.1%)	7 (15.6%)	
Head and neck	11 (16.4%)	20 (18.2%)		17 (25.8%)	7 (15.6%)	
ESCC	14 (20.9%)	6 (5.5%)		11 (16.7%)	5 (11.1%)	
Others^a,b^	13 (19.4%)	35 (31.8%)		12 (18.2%)	17 (37.8%)	
Performance status						
0-1	64 (95.5%)	108 (98.2%)	0.570	64 (97.0%)	44 (97.8%)	1
2-3	3 (4.5%)	2 (1.8%)		2 (3.0%)	1 (2.2%)	
TNM stage						
III	12 (17.9%)	16 (14.5%)	0.702	12 (18.2%)	9 (20.0%)	1
IV	55 (82.1%)	94 (85.5%)		54 (81.8%)	36 (80.0%)	
Liver metastasis	16 (23.9%)	30 (27.3%)	0.747	18 (27.3%)	12 (26.7%)	1
Multiple metastases	22 (32.8%)	51 (46.4%)	0.106	23 (34.8%)	21 (46.7%)	0.293
PD-1/PD-L1 inhibitor						
Pembrolizumab	25 (37.3%)	44 (40.0%)	0.727	25 (37.9%)	18 (40.0%)	0.800
Nivolumab	14 (20.9%)	26 (23.6%)		12 (18.2%)	12 (26.7%)	
Toripalimab	7 (10.4%)	14 (12.7%)		9 (13.6%)	4 (8.9%)	
Others^c,d^	21 (31.3%)	26 (23.6%)		20 (30.3%)	11 (24.4%)	
No prior systemic therapy	41 (61.2%)	57 (51.8%)	0.289	39 (59.1%)	20 (44.4%)	0.185
Combination therapy^e,f^	51 (76.1%)	83 (75.5%)	1	53 (80.3%)	33 (73.3%)	0.528
Elevated serum LDH	16 (23.9%)	20 (18.2%)	0.29	17 (25.8%)	8 (17.8%)	0.428
Immunoglobulin, median (range)						
IgG, g/L	12.1 (8.53-19.10)	11.3 (5.17-19.40)	**0.031**	12.1 (8.53, 19.4)	11.3 (5.17, 18.0)	0.044
IgA, g/L	2.50 (1.24-4.88)	2.33 (0.64-5.71)	0.683	2.51 (0.73, 5.71)	2.27 (0.64, 4.00)	0.234
IgM, g/L	0.925 (0.43-2.20)	0.820 (0.20-2.92)	0.397	0.975 (0.26, 1.96)	0.73 (0.20, 2.92)	0.259
PD-L1 status						
Positive^g^	23 (34.3%)	28 (25.5%)	0.273	19 (28.8%)	11 (24.4%)	0.281
Negative	7 (10.4%)	8 (7.3%)		8 (12.1%)	2 (4.4%)	
Unknown	37 (55.2%)	74 (67.3%)		39 (59.1%)	32 (71.1%)	
MSI status						
MSI-H	6 (9.0%)	7 (6.4%)	0.800	5 (7.6%)	2 (4.4%)	0.763
MSS	15 (22.4%)	24 (21.8%)		14 (21.2%)	11 (24.4%)	
Unknown	46 (68.7%)	79 (71.8%)		47 (71.2%)	32 (71.1%)	
Anti-Ro52 antibody						
Positive	16 (23.9%)	11 (10.0%)	**0.035**	25 (37.9%)	0 (0%)	**<0.001**
Negative	38 (56.7%)	68 (61.8%)		41 (62.1%)	45 (100%)	
Unknown	13 (19.4%)	31 (28.2%)		–	–	
Antithyroid antibody^h^						
Positive	9 (13.4%)	7 (6.4%)	0.065	12 (18.2%)	0 (0%)	**0.0067**
Negative	51 (76.1%)	79 (71.8%)		54 (81.8%)	45 (100%)	
Unknown	7 (10.4%)	24 (21.8%)		–	–	
Any preexisting antibody^i^						
Positive	47 (70.1%)	19 (17.3%)	**<0.001**	**-**	–	–
Negative	0 (0%)	45 (40.9%)		–	–	–
Unknown	20 (29.9%)	46 (41.8%)		–	–	–
Antinuclear antibody						
Positive	–	–	–	47 (71.2%)	0 (0%)	**<0.001**
Negative	–	–	–	19 (28.8%)	45 (100%)	

ANA, antinuclear antibody; ESCC, esophageal cell squamous carcinoma; GC, gastric cancer; LDH, lactate dehydrogenase; MSI, microsatellite instability; MSI-H, MSI-high; MSS, microsatellite-stable; NSCLC, non-small cell lung cancer; PD-L1, programmed death ligand-1.

a For ANA, 16 patients with urological cancer, 14 with colorectal cancer, 3 with cholangiocarcinoma, 3 with pancreatic cancer, 3 with peritoneal mesothelioma, 2 with cervical cancer, 2 with sarcoma, 1 with small cell lung cancer, 1 with gallbladder cancer, 1 with endometrial cancer, 1 with neuroendocrine neoplasm, and 1 with Merkel cell carcinoma.

b For any preexisting antibody, 10 patients with colorectal cancer, 6 with urological cancer, 3 with peritoneal mesothelioma, 2 with pancreatic cancer, 2 with cervical cancer, 1 with sarcoma, 1 with small cell lung cancer, 1 with gallbladder cancer, 1 with cholangiocarcinoma, 1 with neuroendocrine neoplasm, and 1 with Merkel cell carcinoma.

c For ANA, 18 patients treated with tislelizumab, 10 with sintilimab, 8 with camrelizumab, 6 with penpulimab, 3 with durvalumab, and 2 with geptanolimab.

d For any preexisting antibody, 10 patients treated with tislelizumab, 7 with sintilimab, 5 patients treated with camrelizumab, 5 with penpulimab, 3 with durvalumab, and 1 with geptanolimab.

e For ANA, 99 patients treated with combined chemotherapy, 27 with combined targeted therapy, 7 with combined chemotherapy plus targeted therapy, and 1 with combined ipilimumab.

f For any preexisting antibody, 63 patients treated with combined chemotherapy, 17 with combined targeted therapy, and 6 with combined chemotherapy plus targeted therapy.

g PD-L1 combined positive score ≥ 1 or tumor proportion score ≥ 1%.

h The patients were considered positive if either antithyroglobulin or antithyroid peroxidase was positive.

i The patients were considered positive if all autoantibodies including ANA, ANA profile, and antithyroid antibodies were examined and any autoantibody was positive.

As shown in **Table 1** and **Supplementary Table S1**, there was no significant difference in age, sex, PS, TNM stage, treatment line, PD-L1 status, and microsatellite instability (MSI) status between patients with or without preexisting ANA, anti-Ro52, antithyroid antibody, or any antibody. However, positive ANA was associated with higher serum IgG level and was more common in patients with esophageal cell squamous carcinoma.

### Safety Analysis

Eighty-two (46.3%) patients experienced irAEs of any grade, 14 (7.9%) patients developed irAEs of grade 3-5. Specifically, 42 (23.7%) developed skin reactions, 36 (20.3%) developed thyroid dysfunction, whereas 10 (5.6%) developed pneumonitis. These irAEs readily resolved with symptomatic treatments and did not lead to interruption of therapy in most cases. However, 26 (14.7%) patients required systemic immunosuppressants, and 22 (12.4%) patients discontinued immunotherapy. Notably, the timing of irAEs occurrence ranged from 1 day to 2 years following the initiation of PD-1/PD-L1 inhibitors but occurred mainly at 1 to 10 weeks (75/82, 91%).

As shown in **Table 2**, thyroid dysfunction was more frequent in patients with positive antithyroid antibody (75.0% versus 13.8%, p < 0.001). However, the presence of positive ANA, anti-Ro52, or any antibody had no significant association with the development of irAEs of any grade or grades 3-5, and the development of skin reactions and thyroid dysfunction. Moreover, preexisting ANA, anti-Ro52, antithyroid, or any antibody was not correlated with the early emergence of irAEs, systemic immunosuppressant treatments required for irAEs, and immunotherapy discontinuation due to irAEs.

**Table 2:** Development of irAEs and treatment response among patients with or without preexisting autoantibodies.

	Positive ANA	Negative ANA	P value	Positive anti-Ro52	Negative anti-Ro52	P value	Positive antithyroid	Negative antithyroid	P value	Positive for any preexisting antibody	Negative for all preexisting antibodies	P value
	(n=67)	(n=110)		(n=27)	(n=106)		(n=16)	(n=130)		(n=66)	(n=45)	
irAEs												
Any grade	32 (47.8%)	50 (45.5%)	0.886	14 (51.9%)	50 (47.2%)	0.827	14 (87.5%)	53 (40.8%)	**0.001**	34 (51.5%)	18 (40.0%)	0.317
Grades 3–5	4 (6.0%)	10 (9.1%)	0.646	5 (18.5%)	8 (7.5%)	0.177	2 (12.5%)	10 (7.7%)	0.858	6 (9.1%)	5 (11.1%)	0.979
Skin reactions	16 (23.9%)	26 (23.6%)	1	5 (18.5%)	27 (25.5%)	0.615	6 (37.5%)	30 (23.1%)	0.339	14 (21.2%)	13 (28.9%)	0.484
Thyroid dysfunction	17 (25.4%)	19 (17.3%)	0.269	5 (18.5%)	23 (21.7%)	0.922	12 (75.0%)	18 (13.8%)	**<0.001**	19 (28.8%)	6 (13.3%)	0.093
Gap between irAEs occurrence and ICI initiation (days)	39.5 (1-238)	42.5 (3-738)	0.202	40 (3-238)	34.5 (1-738)	0.828	46.5 (1- 126)	36 (2- 738)	0.523	39.5 (1-238)	25 (3-738)	0.596
Systemic immunosuppression required for irAEs	7 (10.4%)	19 (17.3%)	0.305	6 (22.2%)	16 (15.1%)	0.549	3 (18.8%)	17 (13.1%)	0.812	10 (15.2%)	7 (15.6%)	1
ICI discontinuation due to irAEs	6 (9.0%)	16 (14.5%)	0.391	5 (18.5%)	15 (14.2%)	0.791	2 (12.5%)	14 (10.8%)	1	7 (10.6%)	7 (15.6%)	0.631
ORR	31 (46.3%)	36 (32.7%)	0.101	14 (51.9%)	41 (38.7%)	0.307	5 (31.2%)	50 (38.5%)	0.773	32 (48.5%)	14 (31.1%)	0.104
DCR	58 (86.6%)	87 (79.1%)	0.293	26 (96.3%)	83 (78.3%)	0.059	13 (81.2%)	105 (80.8%)	1	57 (86.4%)	32 (71.1%)	0.082

ANA, antinuclear antibody; DCR, disease control rate; ICIs, immune checkpoint inhibitors; irAEs, immune-related adverse events; ORR, objective response rate.

### Evaluation of Efficacy

After receiving PD-1/PD-L1 inhibitors therapy, 4 patients achieved complete response, ORR and DCR in enrolled patients were 37.9% and 81.9%, respectively. As shown in **Table 2**, preexisting ANA, anti-Ro52, antithyroid, or any antibody had no significant influence on ORR and DCR in patients treated with PD-1/PD-L1 inhibitors. However, there was a trend for a higher DCR in those positive for anti-Ro52 antibody than those negative for anti-Ro52 (96.3% versus 78.3%, p = 0.059).

**Figure 1:**
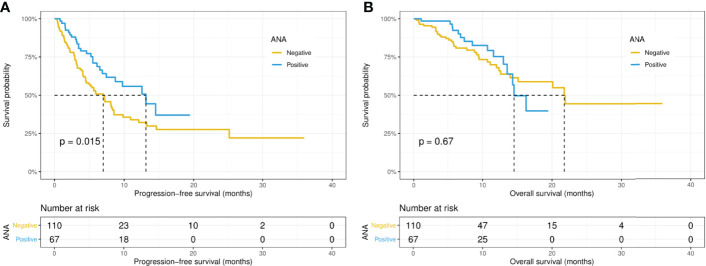
Kaplan-Meier curves of progression-free survival **(A)** and overall survival **(B)** in the positive and negative ANA groups. ANA, antinuclear antibody.

**Figure 2:**
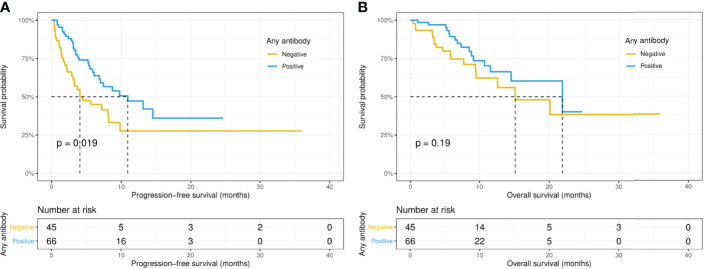
Kaplan-Meier curves of progression-free survival **(A)** and overall survival **(B)** in patients with or without any preexisting antibody.

**Figure 3:**
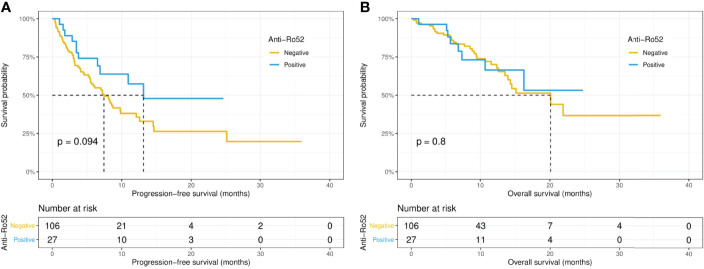
Kaplan-Meier curves of progression-free survival **(A)** and overall survival **(B)** in the positive and negative anti-Ro52 antibody groups.

**Figure 4:**
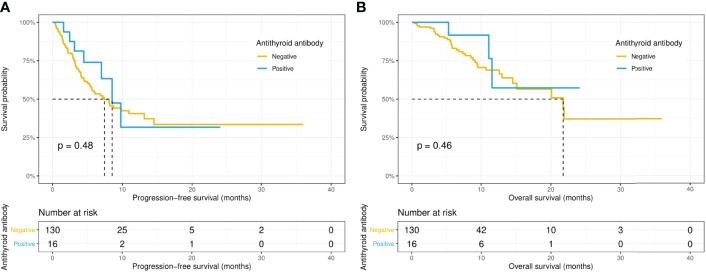
Kaplan-Meier curves of progression-free survival **(A)** and overall survival **(B)** in the positive and negative antithyroid antibody groups.

Intriguingly, the median PFS was significantly longer in the ANA-positive patients (13.1 versus 7.0 months, p = 0.015), while the median OS did not differ significantly (14.5 versus 21.8 months, p = 0.67) between the ANA-positive and ANA-negative groups (**Figures 1A, B**). Similarly, the median PFS was significantly longer in those with any preexisting antibody (10.9 versus 4.1 months, p = 0.019), while the median OS did not differ significantly (21.9 versus 15.1 months, p = 0.19) between those with and without any preexisting antibody (**Figures 2A, B**). With adjusting the impact of confounding factors using PSM analysis, the patients with preexisting ANA, or any antibody had robustly longer PFS, and the OS did not differ significantly (**Supplementary Figures S1**, **2**). However, there were no significant differences in PFS (14.5 versus 8.1 months, p = 0.31) or OS (14.5 versus 21.8 months, p = 0.80) between those with or without ≥ 1:160 ANA titers (**Supplementary Figures S3A, B**). Moreover, the preexisting anti-Ro52 and antithyroid antibodies were not associated with PFS (13.1 versus 7.4 months, p = 0.094; 8.5 versus 7.4 months, p = 0.48, respectively) and OS (not reached versus 20.1 months, p = 0.80; not reached versus 21.8 months, p = 0.46, respectively) (**Figures 3**, **4**).

Considering the sample size and collinearity between variables, we first performed LASSO regression analysis on variables that might affect patient survival outcomes. Variables including age, gender, PS, cancer type, TNM stage, liver metastasis, multiple metastases, treatment line, combination therapy, the level of LDH, IgG, IgA, IgM, IL-6, IL-8, IL-10, TNF-α, hsCRP, ESR, PD-L1 status, MSI status, any preexisting antibody (positive or negative), ANA (positive or negative), ANA titer (≥1:160 or <160), anti-Ro52 antibody (positive or negative), and antithyroid antibody (positive or negative) were examined in LASSO regression. After performing LASSO regression, age, gender, PS, liver metastasis, multiple metastases, treatment line, the level of LDH, MSI status, ANA, and anti-Ro52 antibody were identified as the potential predictors of PFS (**Supplementary Figure S4**), while gender, PS, TNM stage, liver metastasis, multiple metastases, treatment line, the level of LDH, MSI status, and any preexisting antibody were identified as the potential predictors of OS (**Supplementary Figure S5**). Furthermore, as shown in **Table 3**, the univariate analysis demonstrated that preexisting ANA, MSI status, treatment line, liver metastasis, and multiple metastases were significantly associated with the PFS of patients treated by PD-1/PD-L1 inhibitors. Further multivariate analysis confirmed that positive ANA (HR: 0.59, 95% CI: 0.37-0.92, p = 0.021) was an independent indicator of better PFS. However, preexisting autoantibodies were not independently associated with the OS of the patients (**Table 4**).

**Table 3:** Univariate and multivariate analyses of factors for progression-free survival.

Variables	Univariate analysis		Multivariate analysis	
	HR (95% CI)	P value	HR (95% CI)	P value
Age (≥60 vs. <60)	0.7 (0.47,1.05)	0.082	-	-
Sex (Male vs. Female)	1.36 (0.86,2.14)	0.187	-	-
Performance status (2-3 vs. 0-1)	2.03 (0.64,6.43)	0.229	-	-
Liver metastasis (Yes vs. No)	2.98 (1.93,4.58)	**<0.001**	2.69 (1.64,4.43)	**<0.001**
Multiple metastases (Yes vs. No)	2.15 (1.43,3.22)	**<0.001**	1.32 (0.84,2.07)	0.237
No prior systemic therapy (Yes vs. No)	0.54 (0.36,0.81)	**0.003**	0.55 (0.36,0.83)	**0.005**
Elevated LDH (Yes vs. No)	1.37 (0.84,2.24)	0.211	-	-
MSI status (MSI-H vs. MSS)	0.18 (0.05,0.6)	**0.005**	3.34 (0.97,11.43)	0.055
ANA (Positive vs. Negative)	0.58 (0.37,0.91)	**0.017**	0.59 (0.37,0.92)	**0.021**
Anti-Ro52 antibody (Positive vs. Negative)	0.58 (0.3,1.1)	0.097	-	-

ANA, antinuclear antibody; LDH, lactate dehydrogenase; MSI, microsatellite instability; MSI-H, MSI-high; MSS, microsatellite-stable.

**Table 4:** Univariate and multivariate analyses of factors for overall survival.

Variables	Univariate analysis		Multivariate analysis	
	HR (95% CI)	P value	HR (95% CI)	P value
Sex (Male vs. Female)	1.38 (0.73,2.59)	0.324	-	-
Performance status (2-3 vs. 0-1)	6.55 (1.97,21.7)	**0.002**	3.33 (0.91,12.23)	0.07
TNM stage (IV vs. III)	8.36 (1.15,60.73)	**0.036**	5.74 (0.73,44.8)	0.096
Liver metastasis (Yes vs. No)	3.9 (2.22,6.86)	**<0.001**	2.1 (1.02,4.32)	**0.043**
Multiple metastases (Yes vs. No)	2.58 (1.46,4.57)	**0.001**	1.05 (0.54,2.05)	0.881
No prior systemic therapy (Yes vs. No)	0.43 (0.24,0.78)	**0.005**	0.53 (0.28,0.98)	**0.042**
Elevated LDH (Yes vs. No)	2.38 (1.3,4.34)	**0.005**	1.88 (0.97,3.63)	0.06
MSI status (MSI-H vs. MSS)	0.1 (0.01,0.77)	**0.027**	0.13 (0.02,1.09)	0.06
Any preexisting antibody (Positive vs. Negative)	0.64 (0.32,1.27)	0.201	-	-

ANA, antinuclear antibody; LDH, lactate dehydrogenase; MSI, microsatellite instability; MSI-H, MSI-high; MSS, microsatellite-stable.

## Discussion

Although PD-1/PD-L1 inhibitors for cancer immunotherapy are currently in common use in oncology, their safety and efficacy are still unknown for patients with preexisting autoantibodies, which are recognized as biomarkers of autoimmune diseases. Naturally, clinicians would be more concerned about severe and fatal irAEs in patients with potential autoimmune diseases, which occur occasionally in the general population with the use of ICIs (17). In the present study, we evaluated the effect of preexisting autoantibodies on the safety and efficacy of PD-1/PD-L1 inhibitors.

Previous research showed that the presumptive percentage of positive ANA in the general population is 10% to 15% with the cutoff value at 1:80 (18, 19). However, the rate of ANA positivity is even as high as 17% to 51% in patients with cancer (20–22). In the present retrospective cohort, the frequency of ANA positivity was 37.9%. Our results supported that the ANA positivity rate in patients with cancer was higher than that in the general population, but the role of ANA in tumorigenesis and cancer development remains unclear. Preclinical studies suggested ANA has anti-tumor activity (3, 23, 24), ANA positivity in lung cancer patients was reported to be associated with an improved PFS and OS (25), but there were also contrary reports (26).

There were five published studies estimating the effect of ANA on ICIs toxicity and efficacy (5–9). Four of the studies included only patients with NSCLC (5–8). Giannicola et al. (8) reported metastatic NSCLC patients positive for ANA had significantly prolonged PFS and OS, which contradicts the conclusion reached by another study (7). The other two studies (5, 6) suggested that the efficacy and safety of ICIs therapy in patients with NSCLC and positive ANA were comparable to those negative for ANA. Accordingly, there was no significant difference in the incidence of irAEs, ORR, and survival outcome between the 16 NSCLC patients positive for ANA and 30 NSCLC patients negative for ANA retrieved from our cohort (data not shown). Intriguingly, PFS was found to be longer in ANA-positive patients treated by PD-1/PD-L1 inhibitors across tumor types (13.1 versus 7.0 months, p = 0.015), even when the confounding factors (including TNM stage and cancer type) were adjusted. Furthermore, Yoneshima et al. (7) observed the ANA titer increased in 3 patients who were initially positive for ANA, consequently, all of them developed irAEs. However, there was only one patient who developed grade 2 thyroid dysfunction among 7 patients with the increased ANA titer during PD-1 inhibitor treatment. As for the effect of ANA titer, the study of Mouri et al. (5) suggested the incidence of irAEs was not significantly different between the ANA-positive and ANA-negative groups, regardless of the cutoff of ANA titers (1:40 or 1:80). Our results also reached the similar conclusion that patients with preexisting ANA had no increased risk of irAEs, regardless of the cutoff of ANA titers (1:80 or 1:160) (data not shown). Regrettably, there were only 9 patients who had an ANA titer of 1:320 in our cohort, thus, we were unable to analyze the influence of ANA antibody titer ≥ 1:320 on the safety and efficacy of immunotherapy. On the one hand, ANA does not necessarily indicate autoimmune disease, and the general population can also carry ANA, hence, ANA positivity may not represent an increased risk of irAEs. On the other hand, autoantibodies probably are associated with the release of tumor neoantigens (3), ANA positivity is related to immune cells (including NK, T and B cells) activation (3, 27), so ANA-positive patients have a theoretical possibility to achieve better immunotherapy efficacy. These may be the underlying mechanisms for successful immunotherapy in ANA-positive patients in our cohort. However, the association of ANA with anti-tumor immunity needs to be verified by further research.

The rate of anti-Ro52 antibody positivity is about 12% in the general population, and ranges from 5.9% to 30% in cancers (11, 28). However, the presence of anti-Ro52 antibody may not indicate an increased risk of cancer (29). Our data showed the frequency of anti-Ro52 antibody positivity was 20.3% across malignancies, no significant association between cancer type and anti-Ro52 antibody positivity was observed. Furthermore, there was no significant difference in the efficacy and safety of PD-1/PD-L1 inhibitors between patients in the positive anti-Ro52 group and those in the negative group. Notably, there was a trend for better PFS (p = 0.094) in patients positive for anti-Ro52 antibody. Studies with a larger sample size will better clarify the effect of anti-Ro52 antibody on ICIs therapy.

Toi et al. (6) reported NSCLC patients with any preexisting antibody (including ANA, rheumatoid factor, and antithyroid antibody) had significantly better PFS and OS, while no significant differences in PFS and OS were observed between patients with or without preexisting ANA. Nevertheless, in the present study, Kaplan-Meier curves and log-rank tests showed patients with ANA or any preexisting antibody had better PFS, while LASSO and Cox regression analysis demonstrated that only ANA was an independent indicator of better PFS. In addition, our result suggested positive antithyroid antibody was associated with thyroid dysfunction during anti-PD-1/PD-L1 treatment. This is consistent with the conclusion reached by Toi et al. (6).

Our study adds to the growing evidence supporting the use of immunotherapy in patients with preexisting autoantibodies. Moreover, to the best of our knowledge, this is the first study to evaluate the effect of anti-Ro52 antibody on ICIs administration. However, our study has several limitations. First, potential inherent selection bias cannot be excluded using an observational retrospective design. Patients with severe or active autoimmune diseases were underrepresented. Besides, the incidence of diverse irAEs observed in our study might be influenced by monitoring bias. Second, the single-center approach and the relatively small size of a variety of cancer types may limit the generalization of our results to other settings. Third, the titer change of autoantibodies may reflect the change in the immune activation state of the body, but we failed to analyze the effect of the titer change of autoantibodies on efficacy and irAEs induced by ICIs.

## Conclusion

In summary, our data suggested that PD-1/PD-L1 inhibitors could be administered safely and effectively in patients with preexisting autoantibodies but without active autoimmune disease. However, patients positive for antithyroid antibody should monitor closely thyroid dysfunction during anti-PD-1/PD-L1 treatment.

## Data Availability Statement

The raw data supporting the conclusions of this article will be made available by the authors, without undue reservation.

## Ethics Statement

The studies involving human participants were reviewed and approved by The Medical Ethics Committee of Peking Union Medical College Hospital. Written informed consent was not provided because Patients’ consents for participation and publication were waived due to the retrospective design and the deidentified data of this study.

## Author Contributions

LZ, HT, and RG were involved in conceptualization. HT, RG, XX, YW, JZ, SZ, and CB had contributed to data acquisition. HT and RG carried out data analysis and wrote the original draft. LZ and MG critically revised the manuscript. All authors contributed to the article and approved the submitted version.

## Funding

This work was supported by grants from CAMS Innovation Fund for Medical Sciences (No. 2016-I2M-1-001).

## Conflict of Interest

The authors declare that the research was conducted in the absence of any commercial or financial relationships that could be construed as a potential conflict of interest.

## Publisher’s Note

All claims expressed in this article are solely those of the authors and do not necessarily represent those of their affiliated organizations, or those of the publisher, the editors and the reviewers. Any product that may be evaluated in this article, or claim that may be made by its manufacturer, is not guaranteed or endorsed by the publisher.

## Supplementary Material

Supplementary Table 1 Baseline characteristics of patients with or without preexisting anti-Ro52 or antithyroid antibodies. ANA, antinuclear antibody; ESCC, esophageal cell squamous carcinoma; GC, gastric cancer; LDH, lactate dehydrogenase; MSI, microsatellite instability; MSI-H, MSI-high; MSS, microsatellite-stable; NSCLC, non-small cell lung cancer; PD-L1, programmed death ligand-1. ^a^ For anti-Ro52 antibody, 12 patients with colorectal cancer, 6 with urological cancer, 3 with peritoneal mesothelioma, 2 with pancreatic cancer, 2 with cervical cancer, 2 with sarcoma, 1 with small cell lung cancer, 1 with cholangiocarcinoma, 1 with gallbladder cancer, 1 with neuroendocrine neoplasm, and 1 with Merkel cell carcinoma. ^b^ For antithyroid antibody, 12 patients with urological cancer, 11 with colorectal cancer, 3 with cholangiocarcinoma, 3 with pancreatic cancer, 3 with peritoneal mesothelioma, 2 with cervical cancer, 1 with sarcoma, 1 with small cell lung cancer, 1 with gallbladder cancer, 1 with neuroendocrine neoplasm, and 1 with Merkel cell carcinoma. ^c^ For anti-Ro52 antibody, 12 patients treated with tislelizumab, 8 with sintilimab, 5 with camrelizumab, 5 with penpulimab, 3 with durvalumab, and 2 with geptanolimab. ^d^ For antithyroid antibody, 13 patients treated with tislelizumab, 8 with sintilimab, 8 with camrelizumab, 6 with penpulimab, 3 with durvalumab, and 1 with geptanolimab. ^e^ PD-L1 combined positive score ≥ 1 or tumor proportion score ≥ 1%. ^f^ The patients were considered positive if all autoantibodies including ANA, ANA profile, and antithyroid antibodies were examined and any autoantibody was positive. ^g^ The patients were considered positive if either antithyroglobulin or antithyroid peroxidase was positive.

Supplementary Figure 1 Kaplan-Meier curves of progression-free survival **(A)** and overall survival **(B)** in the positive and negative ANA groups with the adjustment of the impact of confounding factors using propensity-score matching analysis. ANA, antinuclear antibody.

Supplementary Figure 2 Kaplan-Meier curves of progression-free survival **(A)** and overall survival **(B)** in patients with or without any preexisting antibody with the adjustment of the impact of confounding factors using propensity-score matching analysis.

Supplementary Figure 3 Kaplan-Meier curves of progression-free survival **(A)** and overall survival **(B)** in the patients with or without ≥ 1:160 ANA titers. ANA, antinuclear antibody.

Supplementary Figure 4 Identification of the potential predictors of progression-free survival. **(A)** LASSO coefficient profiles of the selected variables. **(B)** Parameter selection in the LASSO model. LASSO, least absolute shrinkage and selection operator.

Supplementary Figure 5 Identification of the potential predictors of overall survival. **(A)** LASSO coefficient profiles of the selected variables. **(B)** Parameter selection in the LASSO model. LASSO, least absolute shrinkage and selection operator.
