# Supplementary material for: Safety and Efficacy of PD-1/PD-L1 Inhibitors in Cancer Patients With Preexisting Autoantibodies
Source: Front Immunol. 2022 May 16;13:893179. doi: 10.3389/fimmu.2022.893179 (PMC9148956; doi:10.3389/fimmu.2022.893179)
Supplement: Supplementary Table 1 — Baseline characteristics of patients with or without preexisting anti-Ro52 or antithyroid antibodies. ANA, antinuclear antibody; ESCC, esophageal cell squamous carcinoma; GC, gastric cancer; LDH, lactate dehydrogenase; MSI, microsatellite instability; MSI-H, MSI-high; MSS, microsatellite-stable; NSCLC, non-small cell lung cancer; PD-L1, programmed death ligand-1. a For anti-Ro52 antibody, 12 patients with colorectal cancer, 6 with urological cancer, 3 with peritoneal mesothelioma, 2 with pancreatic cancer, 2 with cervical cancer, 2 with sarcoma, 1 with small cell lung cancer, 1 with cholangiocarcinoma, 1 with gallbladder cancer, 1 with neuroendocrine neoplasm, and 1 with Merkel cell carcinoma. b For antithyroid antibody, 12 patients with urological cancer, 11 with colorectal cancer, 3 with cholangiocarcinoma, 3 with pancreatic cancer, 3 with peritoneal mesothelioma, 2 with cervical cancer, 1 with sarcoma, 1 with small cell lung cancer, 1 with gallbladder cancer, 1 with neuroendocrine neoplasm, and 1 with Merkel cell carcinoma. c For anti-Ro52 antibody, 12 patients treated with tislelizumab, 8 with sintilimab, 5 with camrelizumab, 5 with penpulimab, 3 with durvalumab, and 2 with geptanolimab. d For antithyroid antibody, 13 patients treated with tislelizumab, 8 with sintilimab, 8 with camrelizumab, 6 with penpulimab, 3 with durvalumab, and 1 with geptanolimab. e PD-L1 combined positive score ≥ 1 or tumor proportion score ≥ 1%. f The patients were considered positive if all autoantibodies including ANA, ANA profile, and antithyroid antibodies were examined and any autoantibody was positive. g The patients were considered positive if either antithyroglobulin or antithyroid peroxidase was positive. [file Table_1.docx]

Table S1. Baseline characteristics of patients with or without preexisting anti-Ro52 or antithyroid antibodies.

| **Variables** | **Positive anti-Ro52** | **Negative anti-Ro52** | **P value** | **Positive antithyroid** | **Negative antithyroid** | **P value** |
| --- | --- | --- | --- | --- | --- | --- |
|  | **(n=27)** | **(n=106)** |  | **(n=16)** | **(n=130)** |  |
| Age, median (range), years | 59 (32, 76) | 60 (22, 85) | 0.838 | 59 (36, 75) | 61 (32, 85) | 0.955 |
| Sex, male | 19 (70.4%) | 75 (70.8%) | 1 | 10 (62.5%) | 96 (73.8%) | 0.507 |
| Tumor type |  |  |  |  |  |  |
| NSCLC | 9 (33.3%) | 27 (25.5%) | 0.368 | 4 (25.0%) | 32 (24.6%) | 0.353 |
| GC | 2 (7.4%) | 19 (17.9%) |  | 2 (12.5%) | 24 (18.5%) |  |
| Head and neck | 7 (25.9%) | 19 (17.9%) |  | 6 (37.5%) | 21 (16.2%) |  |
| ESCC | 5 (18.5%) | 13 (12.3%) |  | 2 (12.5%) | 16 (12.3%) |  |
| Others ^a, b^ | 4 (14.8%) | 28 (26.4%) |  | 2 (12.5%) | 37 (28.5%) |  |
| Performance status |  |  |  |  |  |  |
| 0-1 | 27 (100%) | 102 (96.2%) | 0.694 | 16 (100%) | 126 (96.9%) | 1 |
| 2-3 | 0 (0%) | 4 (3.8%) |  | 0 (0%) | 4 (3.1%) |  |
| TNM stage |  |  |  |  |  |  |
| III | 5 (18.5%) | 19 (17.9%) | 1 | 2 (12.5%) | 23 (17.7%) | 0.866 |
| IV | 22 (81.5%) | 87 (82.1%) |  | 14 (87.5%) | 107 (82.3%) |  |
| Liver metastasis | 6 (22.2%) | 29 (27.4%) | 0.767 | 4 (25.0%) | 35 (26.9%) | 1 |
| Multiple metastases | 10 (37.0%) | 42 (39.6%) | 0.980 | 4 (25.0%) | 58 (44.6%) | 0.219 |
| PD-1/PD-L1 inhibitor |  |  |  |  |  |  |
| Pembrolizumab | 8 (29.6%) | 42 (39.6%) | 0.234 | 4 (25.0%) | 54 (41.5%) | 0.22 |
| Nivolumab | 7 (25.9%) | 25 (23.6%) |  | 3 (18.8%) | 29 (22.3%) |  |
| Toripalimab | 6 (22.2%) | 10 (9.4%) |  | 1 (6.2%) | 16 (12.3%) |  |
| Others ^c, d^ | 6 (22.2%) | 29 (27.8%) |  | 8 (50.0%) | 31 (23.8%) |  |
| No prior systemic therapy | 17 (63.0%) | 53 (50.0%) | 0.323 | 8 (50.0%) | 73 (56.2%) | 0.841 |
| Combination therapy | 21 (77.8%) | 81 (76.4%) | 1 | 13 (81.2%) | 101 (77.7%) | 0.997 |
| Elevated serum LDH | 11 (40.7%) | 17 (16.0%) | 0.019 | 3 (18.8%) | 28 (21.5%) | 0.881 |
| Immunoglobulin, median (range) |  |  |  |  |  |  |
| IgG, g/L | 12.0 (8.97, 19.4) | 11.5 (5.17, 19.1) | 0.164 | 12.7 (9.27, 15.0) | 11.3 (5.17, 19.4) | 0.061 |
| IgA, g/L | 2.24 (0.73, 5.71) | 2.42 (0.64, 4.88) | 0.48 | 2.56 (1.24, 3.74) | 2.34 (0.64, 5.71) | 0.987 |
| IgM, g/L | 0.915 (0.26, 1.55) | 0.845 (0.20, 2.92) | 0.342 | 1.17 (0.50, 1.57) | 0.77 (0.20, 2.92) | 0.04 |
| PD-L1 status |  |  |  |  |  |  |
| Positive ^e^ | 5 (18.5%) | 31 (29.2%) | 0.224 | 5 (31.2%) | 40 (30.8%) | **0.049** |
| Negative | 1 (3.7%) | 11 (10.4%) |  | 4 (25.0%) | 9 (6.9%) |  |
| Unknown | 21 (77.8%) | 64 (60.4%) |  | 7 (43.8%) | 81 (62.3%) |  |
| MSI status |  |  |  |  |  |  |
| MSI-H | 2 (7.4%) | 6 (5.7%) | 0.78 | 0 (0%) | 10 (7.7%) | 0.231 |
| MSS | 5 (18.5%) | 26 (24.5%) |  | 2 (12.5%) | 32 (24.6%) |  |
| Unknown | 20 (74.1%) | 74 (69.8%) |  | 14 (87.5%) | 88 (67.7%) |  |
| Any preexisting antibody ^f^ |  |  |  |  |  |  |
| Positive | 25 (92.6%) | 41 (38.7%) | **<0.001** | 12 (75.0%) | 54 (41.5%) | **0.011** |
| Negative | 0 (0%) | 45 (42.5%) |  | 0 (0%) | 45 (34.6%) |  |
| Unknown | 2 (7.4%) | 20 (18.9%) |  | 4 (25.0%) | 31 (23.8%) |  |
| Antinuclear antibody |  |  |  |  |  |  |
| Positive | 16 (59.3%) | 38 (35.8%) | **0.046** | 9 (56.2%) | 51 (39.2%) | 0.3 |
| Negative | 11 (40.7%) | 68 (64.2%) |  | 7 (43.8%) | 79 (60.8%) |  |
| Antithyroid antibody ^g^ |  |  |  |  |  |  |
| Positive | 3 (11.1%) | 10 (9.4%) | 0.408 | - | - | - |
| Negative | 22 (81.5%) | 77 (72.6%) |  | - | - | - |
| Unknown | 2 (7.4%) | 19 (17.9%) |  | - | - | - |
| Anti-Ro52 antibody | - | - | - | 3 (18.8%) | 22 (16.9%) | 0.899 |
| Positive | - | - | - | 10 (62.5%) | 77 (59.2%) |  |
| Negative | - | - | - | 3 (18.8%) | 31 (23.8%) |  |

ANA, antinuclear antibody; ESCC, esophageal cell squamous carcinoma; GC, gastric cancer; LDH, lactate dehydrogenase; MSI, microsatellite instability; MSI-H, MSI-high; MSS, microsatellite-stable; NSCLC, non-small cell lung cancer; PD-L1, programmed death ligand-1.

^a^ For anti-Ro52 antibody, 12 patients with colorectal cancer, 6 with urological cancer, 3 with peritoneal mesothelioma, 2 with pancreatic cancer, 2 with cervical cancer, 2 with sarcoma, 1 with small cell lung cancer, 1 with cholangiocarcinoma, 1 with gallbladder cancer, 1 with neuroendocrine neoplasm, and 1 with Merkel cell carcinoma.

^b^ For antithyroid antibody, 12 patients with urological cancer, 11 with colorectal cancer, 3 with cholangiocarcinoma, 3 with pancreatic cancer, 3 with peritoneal mesothelioma, 2 with cervical cancer, 1 with sarcoma, 1 with small cell lung cancer, 1 with gallbladder cancer, 1 with neuroendocrine neoplasm, and 1 with Merkel cell carcinoma.

^c^ For anti-Ro52 antibody, 12 patients treated with tislelizumab, 8 with sintilimab, 5 with camrelizumab, 5 with penpulimab, 3 with durvalumab, and 2 with geptanolimab.

^d^ For antithyroid antibody, 13 patients treated with tislelizumab, 8 with sintilimab, 8 with camrelizumab, 6 with penpulimab, 3 with durvalumab, and 1 with geptanolimab.

^e^ PD-L1 combined positive score ≥ 1 or tumor proportion score ≥ 1%.

^f^ The patients were considered positive if all autoantibodies including ANA, ANA profile, and antithyroid antibodies were examined and any autoantibody was positive.

^g^ The patients were considered positive if either antithyroglobulin or antithyroid peroxidase was positive
