# Supplementary figures and images for: Safety and Efficacy of PD-1/PD-L1 Inhibitors in Cancer Patients With Preexisting Autoantibodies
Source: Front Immunol. 2022 May 16;13:893179. doi: 10.3389/fimmu.2022.893179 (PMC9148956; doi:10.3389/fimmu.2022.893179)

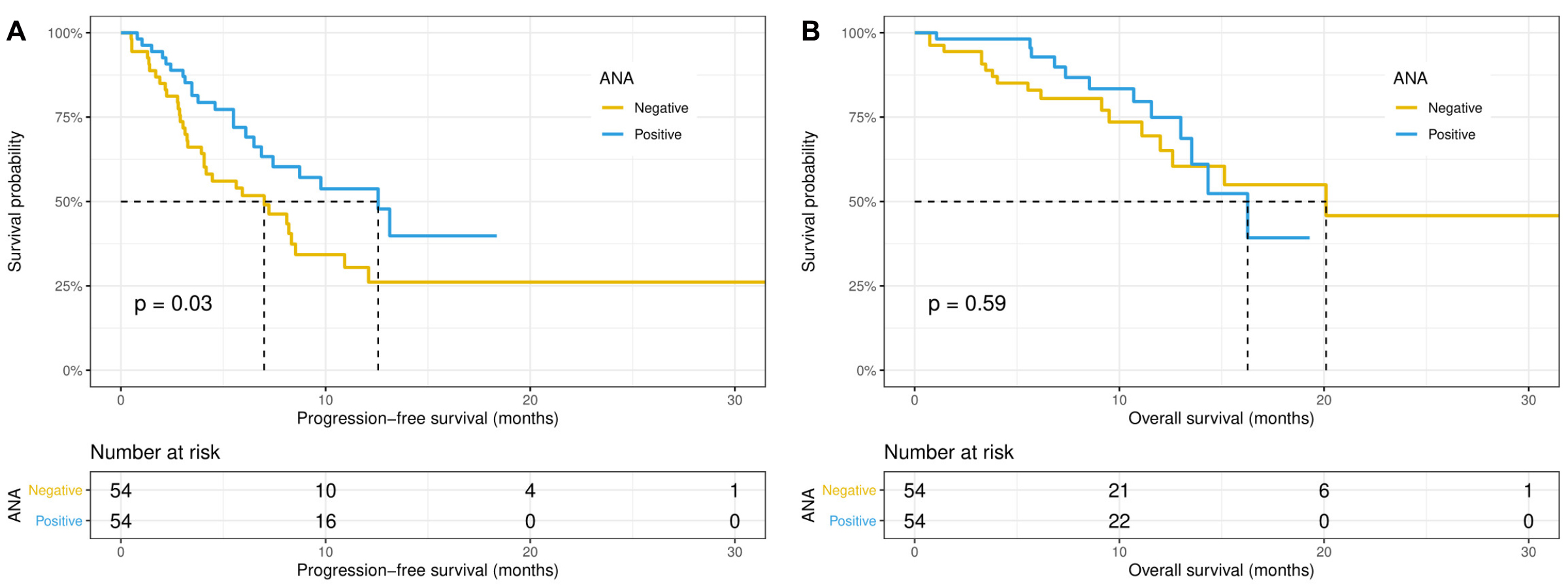

Supplement: Supplementary Figure 1 — Kaplan-Meier curves of progression-free survival (A) and overall survival (B) in the positive and negative ANA groups with the adjustment of the impact of confounding factors using propensity-score matching analysis. ANA, antinuclear antibody. [file Image_1.jpg]

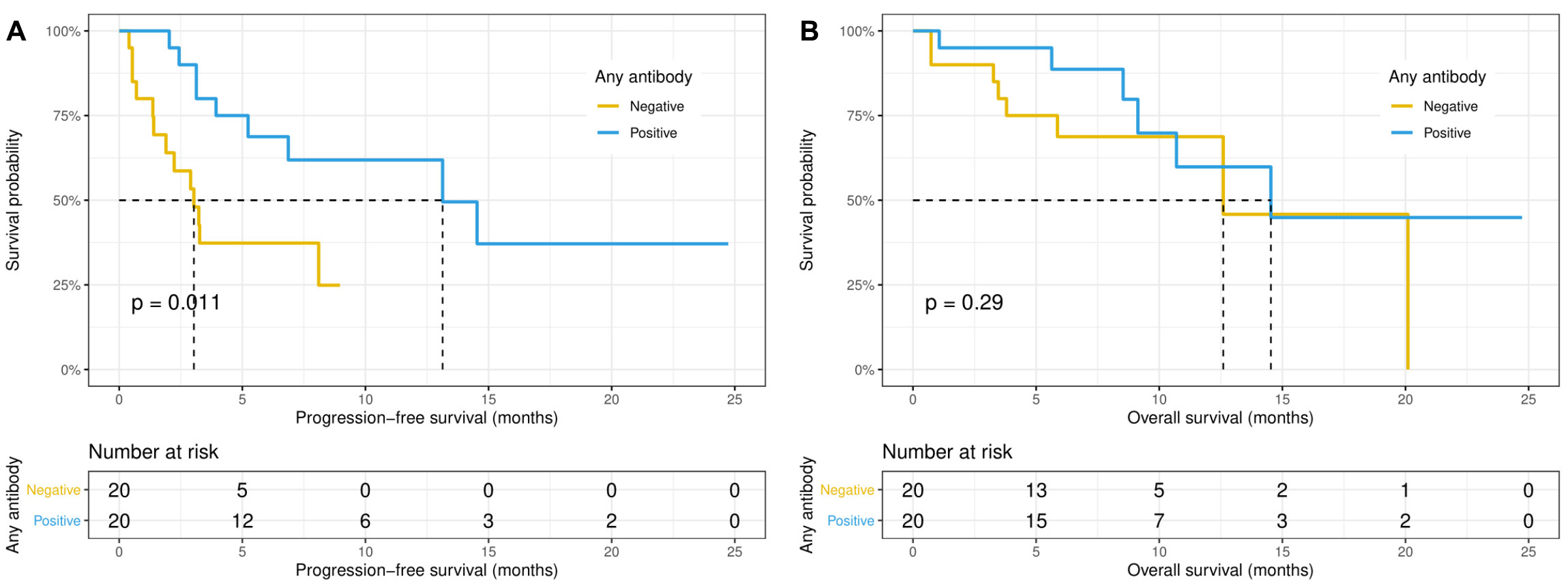

Supplement: Supplementary Figure 2 — Kaplan-Meier curves of progression-free survival (A) and overall survival (B) in patients with or without any preexisting antibody with the adjustment of the impact of confounding factors using propensity-score matching analysis. [file Image_2.jpg]

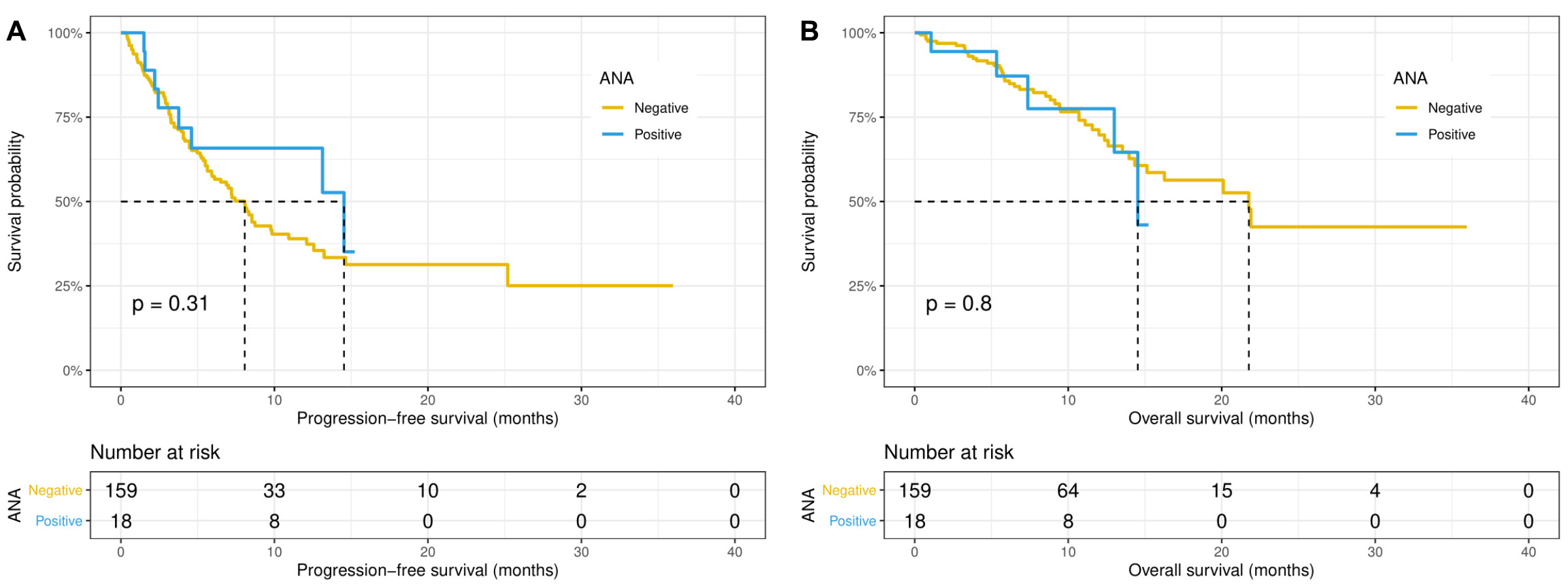

Supplement: Supplementary Figure 3 — Kaplan-Meier curves of progression-free survival (A) and overall survival (B) in the patients with or without ≥ 1:160 ANA titers. ANA, antinuclear antibody. [file Image_3.jpg]

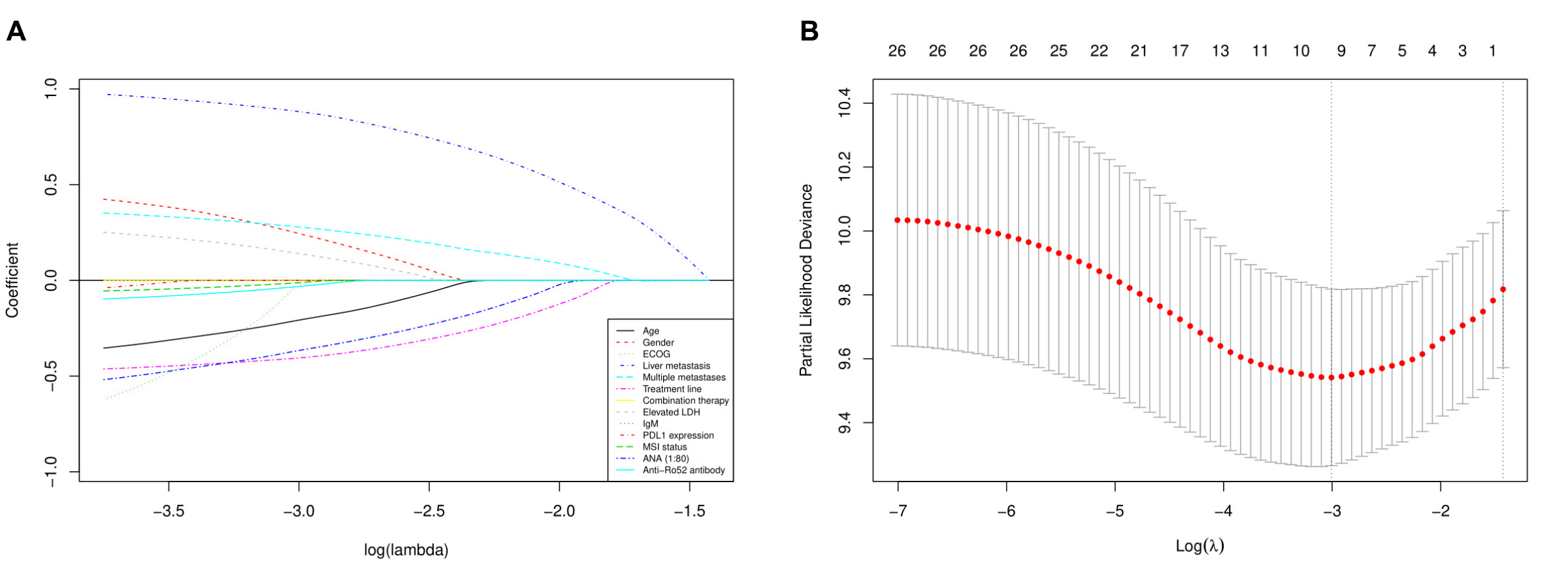

Supplement: Supplementary Figure 4 — Identification of the potential predictors of progression-free survival. (A) LASSO coefficient profiles of the selected variables. (B) Parameter selection in the LASSO model. LASSO, least absolute shrinkage and selection operator. [file Image_4.jpg]

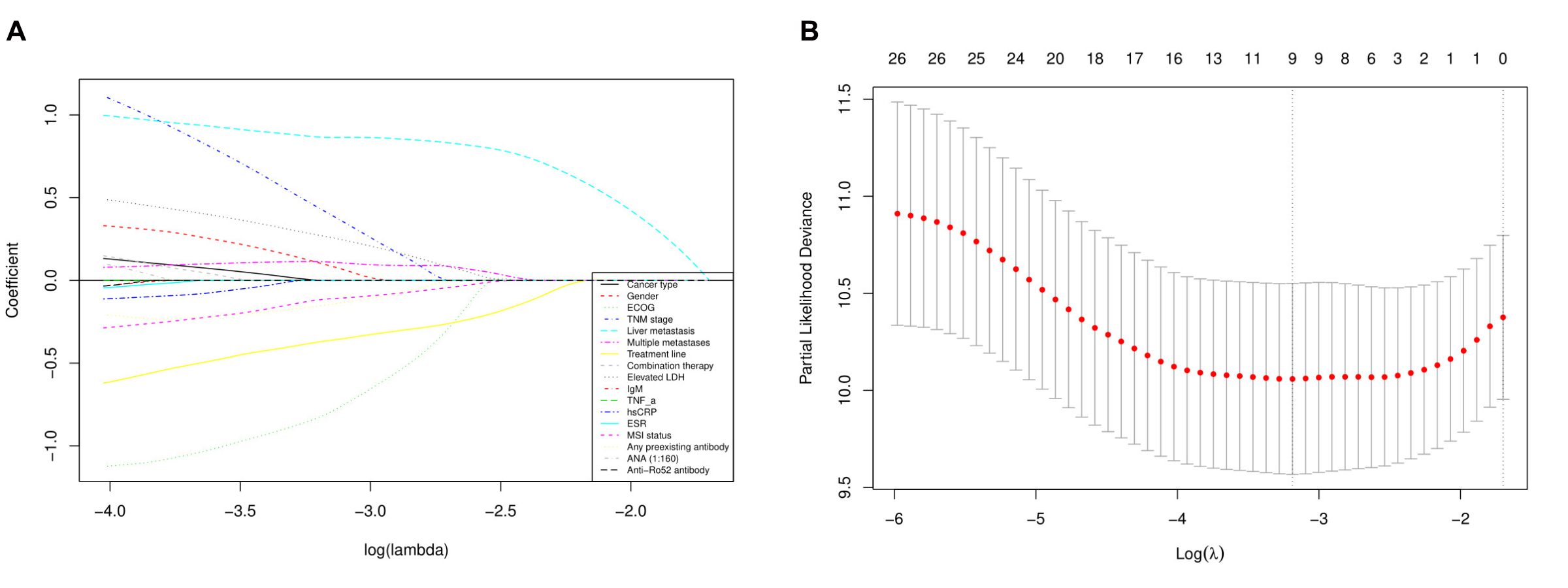

Supplement: Supplementary Figure 5 — Identification of the potential predictors of overall survival. (A) LASSO coefficient profiles of the selected variables. (B) Parameter selection in the LASSO model. LASSO, least absolute shrinkage and selection operator. [file Image_5.jpg]
